# Scalable Microfabrication Procedures for Adhesive-Integrated Flexible and Stretchable Electronic Sensors

**DOI:** 10.3390/s150923459

**Published:** 2015-09-16

**Authors:** Dae Y. Kang, Yun-Soung Kim, Gladys Ornelas, Mridu Sinha, Keerthiga Naidu, Todd P. Coleman

**Affiliations:** Department of Bioengineering, University of California San Diego, La Jolla, CA 92093, USA; E-Mails: dykang@eng.ucsd.edu (D.Y.K.); ysk032@eng.ucsd.edu (Y.-S.K.); glornela@ucsd.edu (G.O.); mbsinha@eng.ucsd.edu (M.S.); agihtreek@gmail.com (K.N.)

**Keywords:** flexible electronics, roll-to-roll, peel-off, flexible sensors

## Abstract

New classes of ultrathin flexible and stretchable devices have changed the way modern electronics are designed to interact with their target systems. Though more and more novel technologies surface and steer the way we think about future electronics, there exists an unmet need in regards to optimizing the fabrication procedures for these devices so that large-scale industrial translation is realistic. This article presents an unconventional approach for facile microfabrication and processing of adhesive-peeled (AP) flexible sensors. By assembling AP sensors on a weakly-adhering substrate in an inverted fashion, we demonstrate a procedure with 50% reduced end-to-end processing time that achieves greater levels of fabrication yield. The methodology is used to demonstrate the fabrication of electrical and mechanical flexible and stretchable AP sensors that are peeled-off their carrier substrates by consumer adhesives. In using this approach, we outline the manner by which adhesion is maintained and buckling is reduced for gold film processing on polydimethylsiloxane substrates. In addition, we demonstrate the compatibility of our methodology with large-scale post-processing using a roll-to-roll approach.

## 1. Introduction

Flexible and stretchable electronics systems—comprised of sensors and circuitry—are increasingly deemed relevant to the future of industrial and consumer electronics devices. The evolution of bulky and rigid electronics into their thin and unobtrusive counterparts has required innovative techniques going beyond standard implementations of CMOS microfabrication. Of these, screen-printing techniques achieve low-cost and scalable processing of flexible sensors and systems [1,2,3,4,5,6,7], though some devices lack the potential for fully integrated electronics with ultra-thin profiles. Engineering of thin film nanocomposites is another example of this trend in miniaturizing our every-day electronic devices [8,9,10,11,12,13,14,15,16,17,18,19,20,21,22,23,24,25,26,27]. Within this class, electronics systems utilizing sacrificial layers (e.g., poly(acrylic acid) (PAA), poly(sodium 4-styrene sulfonate) (PSSNa), poly(n-vinylpyrrolidone) (PVP), poly(methyl methacrylate) (PMMA), water soluble tape, Silicon (Si), SiO_2_) [28,29,30,31] and intermediate substrates (e.g., polydimethylsiloxane (PDMS), water soluble tapes) for transfer printing have allowed for nanomembranes with mechanically-tuned properties [8,9,10,11,12,13,14,15,16,17,32]. For example, epidermal electronics systems boast ultra-thin, high resolution, “skin-like” sensors and circuitry designed for conformal lamination onto the skin [8,9,10,11,12,14,15,16]. These systems excel in the area of intimate integration with contoured and elastic real estates, as compared to traditionally rigid or thin film sheet devices that are otherwise too inflexible and/or unstretchable. The result is robust skin-electrode contact yielding prolonged biosignal acquisition with reduced motion artifact [33]. Overall, these electronics present many significant advances for mobile technologies, but the manner in which they are fabricated requires intermediate materials, and precision involved in transfer printing in order to produce high yields.

A demand for tomorrow’s inconspicuous sensors is quickly rising, in particular due to the advent of “wearable” devices and the Internet of Things (IoT)—a paradigm emphasizing data interconnectedness through the omnipresence of networked sensors and systems. This is exemplified by the five-fold increase in sensors from 2012, resulting in 23.6 billion sensors in 2014 [34]. Unfortunately, these numbers are accompanied by the high cost and complexity of fabricating minimally obtrusive sensors, which proves to be a barrier for widespread adoption of IoT practices in spaces such as healthcare, home, and industrial use [34,35].

Herein we describe an alternative microfabrication approach requiring approximately half the steps of previously reported multi-step transfer printing approaches to build adhesive-integrated flexible and stretchable electrical and mechanical sensors. This reduction in steps has multiple benefits including higher yield and the elimination of time-consuming steps, allowing new opportunities for large-scale production of these sensors. This new approach uses a weakly-adhering interface and inverted fabrication scheme, which obviate the need of a sacrificial layer or intermediate transfer printing, and allow for direct integration of the sensor from the donor substrate to target receiving adhesive. Direct integration without transfer printing is facilitated by a simple mechanical peel-off step—similar in nature to the methods used for exfoliation of graphene and other materials [36,37,38,39], except that a complete device is peeled and packaged without requiring further microfabrication steps. Adhesive-peeled (AP) mechanical strain sensors are demonstrated to maintain their design and connection during axial loading. Moreover, electrical AP sensors are used to record electrophysiological signals with results compared to those of a transfer printing approach [8,9,10,11,12,13], demonstrating similar sensor fidelity while emphasizing a simple peel-off technique that has potential for industry-scale roll-to-roll post-processing.

## 2. Experimental Section

### Process Description

Throughout this discussion, we consider approaches that result in a sensor of pre-specified geometry to be embedded within a given adhesive. The purpose of this work is to facilitate a process by which the same or similar sensor can be made more easily and with higher yield. We compare an approach that utilizes transfer printing (TP) of passive sensors to a new methodology presented herein. To differentiate the two, sensors produced by the TP approach are designated as “TP sensors”, while the presented approach yields “AP sensors”.

**Figure 1:**
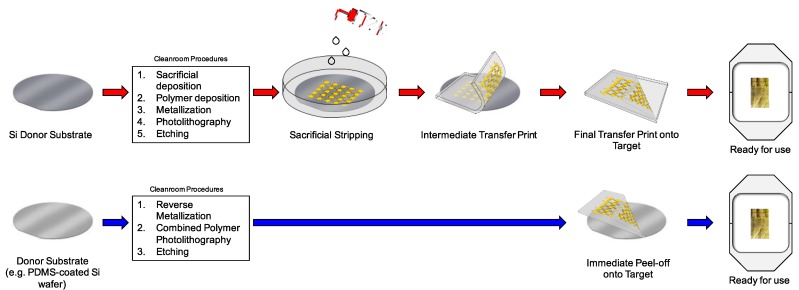
Example of TP *vs.* AP processes. (**Top**) TP process steps, starting with Si donor substrate. Includes procedures such as depositing a sacrificial layer, using solvent for stripping sacrificial layer, and transfer printing by an intermediate elastomeric substrate; (**Bottom**) AP process obviates the need for sacrificial layer, use of solvents, or use of an intermediate elastomer for transfer printing. Instead, a weakly-adhering donor is used, from which reversed metallization and a combined polymer deposition and photolithography step are implemented to create an inverted sensor. Because the sensor is inverted on a weakly-adhering surface, peel-off is performed directly by adhesive. Both processes result in a similar sensor embedded within adhesive; the latter requires fewer steps and materials.

Figure 1 provides a conceptual description of the two approaches. The TP process (top) begins with a donor substrate and undergoes a string of standard cleanroom procedures, which includes depositing a “sacrificial layer” (e.g., PMMA). The donor substrate is then dipped in solvent (e.g., acetone) that dissolves the sacrificial layer and separates the carrier substrate from the other deposited materials. Subsequently, two transferring steps ensue through which the desired pattern is delivered to an intermediate transfer material (e.g., PDMS), and then upon pressure, finally onto a target receiving substrate. This process path culminates in an adhesive with a patterned metal-polymer stack ready for use. The AP process (bottom) obviates the need for depositing a sacrificial layer or intermediate transfer printing and allows for direct application of the adhesive onto the donor substrate to embed the pattern within the adhesive. The AP process accomplishes this reduction in steps and direct transfer to the adhesive through the use of (1) a weakly-adhering donor substrate and (2) inverted production of the metal-polymer stacks comprising the sensor designed. The rationale for this approach will be elucidated in the Discussion section.

**Figure 2:**
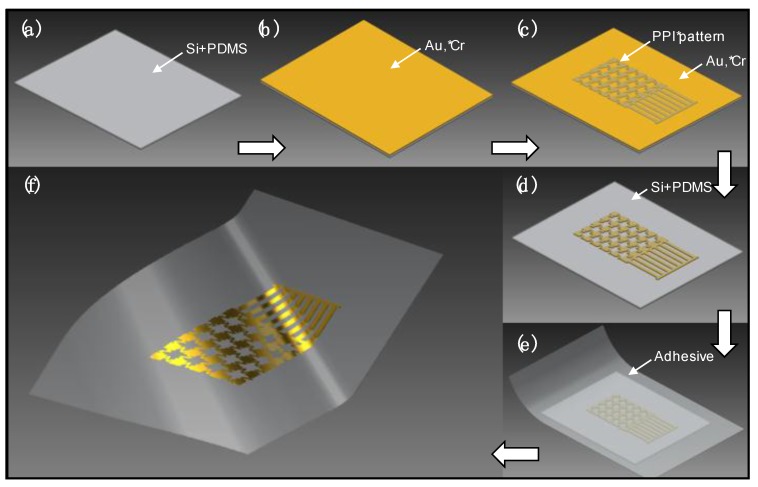
Fabrication process for electrical AP sensors: (**a**) formation of weakly-adhering donor; (**b**) metallization of Au and Cr films, respectively; (**c**) formation and patterning of PPI polymer backbone; (**d**) etch-back of Cr and Au films using PPI as etch mask; (**e**) flexible adhesive substrate adhered to pattern, ready for peel-off; and (**f**) pattern peeled-off onto receiving adhesive with Au surface exposed, ready for use.

The process for fabrication of AP sensors is illustrated in Figure 2. Experimental details can be found in the Supplementary Information under the Experimental Details section. An example for results in this narrative is as follows. A standard Si wafer is coated with a thin silicone layer, creating a weakly-adhering interface, which comprises the donor substrate (Figure 2a). Standard cleanroom techniques are applied in a semi-reversed order for creating inverted flexible sensors on the donor substrate. Sequential metallization of gold (Au) and chromium (Cr) thin films onto PDMS form the conductive layer of these sensors (Figure 2b): the Au thin film interfaces with the weakly-adhering donor while Cr is exposed at the top. A photodefinable polyimide (PPI) is then used for simultaneous formation and patterning of the sensor’s polymer backing (Figure 2c). An etch-back of the exposed Cr-Au regions (Figure 2d) results in a donor substrate with electrical AP sensors that can be directly peeled-off by the adhesive of choice (Figure 2e,f).

## 3. Results

### 3.1. Qualitative Comparison of Fabrication Methods for TP and AP Sensors

**Figure 3:**
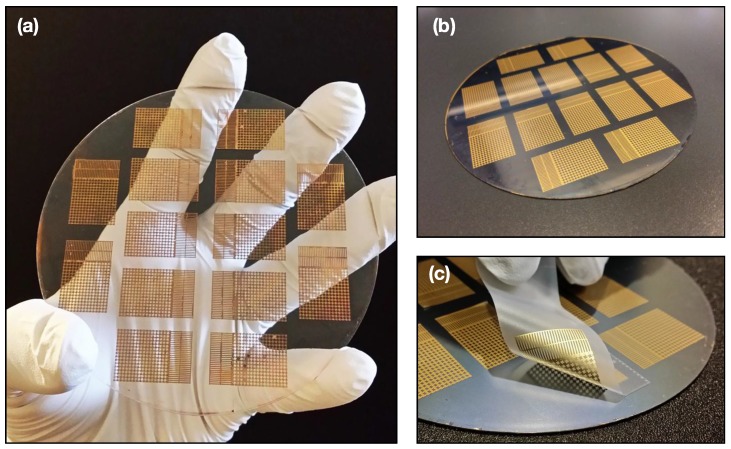
Alternative approach—electrical AP sensors: (**a**) dense sensor assembly on rigid glass donor substrate; (**b**) similar sensor assembly on Si wafer; and (**c**) AP sensor peeled-off from Si donor substrate onto adhesive.

Figure 3 showcases electrical sensors fabricated using the AP process. AP sensors can be densely packed onto the same working area of the donor substrate since submersion in the solvent and wafer dicing are not necessary. Figure 3a shows AP sensors on a four-inch glass wafer, while Figure 3b illustrates AP sensors on a Si wafer. The ease with which a sensor can be peeled-off onto an adhesive material (Scotch Tape, 3M, Saint Paul, MN, USA) is shown (Figure 3c). In contrast, TP sensors are subject to a lengthy release process involving sacrificial layer stripping via solvent treatment, after which a TP sensor carefully undergoes a two-step transfer printing process (Video S1 is shown with sacrificial layer being PMMA, solvent being acetone, and transfer stamp being PDMS—27 minutes). The requirement of controlled pattern release rates from the donor substrate during sacrificial stripping can often lead to sensors that are deformed from the intended design. These challenges are only magnified when considering larger, more complex heterogeneous sensor patterns, often requiring subjective user judgment and manual dexterity.

Conversely, with the AP approach, the desired pattern is fabricated and objectively post-processed onto a receiving adhesive of choice with a one-step peel-off, obviating the need for solvents and intermediate stamps (Video S2—35 seconds). To further demonstrate this—and the possibility of roll-to-roll post-processing—we demonstrate peeling off AP sensors with a consumer-grade lint roller (Video S3). The force required to successfully peel-off AP sensors from the weakly-adhering donor was calculated at 0.22 ± 0.03 N. Experimental details can be found in the Materials and Methods.

**Figure 4:**
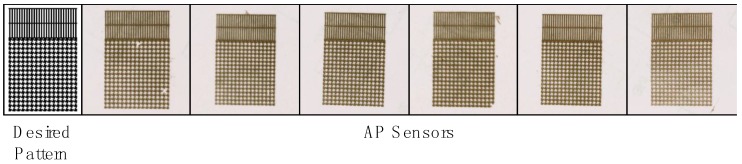
AP Sensor variability: (**Left**) desired sensor pattern and (**Right**) AP sensors consecutively peeled-off from same donor substrate. On average, AP sensors closely reflect the desired pattern, primarily because release rates in solvents pertaining to removal of sacrificial layer are not required.

A montage of consecutively peeled sensors demonstrates the low variability with which electrical sensors are produced using the AP process (Figure 4). Fabricated sensors are accurate to the desired pattern with very little deformity. Although it is possible with the TP approach to retrieve sensors identical to the desired pattern, considerable time and expertise are required if performed manually. Though automation has been demonstrated for TP production of thin film silicon-based electronics [40,41,42], these systems require high precision, multi-point calibration, and visual inspection of transfer printing to ensure high yield post-processing. Because the AP approach requires fewer procedures, does not require sacrificial stripping or undercutting, and is not subject to the variability in release rates, there is significant potential for automated post-processing of AP sensors. Comparing both methods, there is an approximately *50% reduction* in processing time using the AP method.

### 3.2. Fabricating on PDMS Surfaces

Microfabrication on PDMS is expected to yield poor adhesion at PDMS–metal interfaces, due to low wettability of PDMS and the large mismatch in thermal expansion coefficients between elastomers like PDMS and thin metal films [43,44,45]. Consequently, compressive stress develops in thin metal films that induce spontaneous wrinkling and buckling, which can progress to propagating cracks throughout the metal layers [46,47]. However, there are ways to address the issues of premature buckling of thin Au films during processing on PDMS. During fabrication, we found stiffening of the PDMS-based weakly-adhering layer (by reducing the ratio of PDMS pre-polymer to crosslinker) helped prevent adverse buckling during processing. During sputter-based processing, the known formation of a silica-like layer through plasma exposure prevented the delamination of sputtered AP sensors from their weakly-adhering donor substrates.

**Figure 5:**
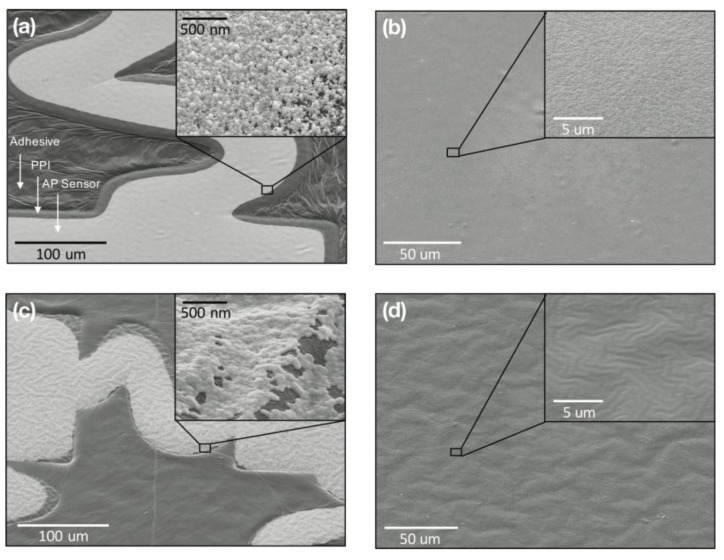
SEM images of evaporated and sputter-coated AP sensors: (**a**) evaporated sensor on adhesive film; (**b**) weakly-adhering PDMS surface after evaporated sensor peel-off; (**c**) sputter-coated sensor on adhesive film; (**d**) weakly-adhering PDMS surface after sputter-coated sensor peel-off; and (**insets**) Zoomed-in surface definition of respective surfaces.

Figure 5 gives a closer look at the surface topography of peeled-off AP sensors and their respective optimized PDMS surfaces for ebeam evaporated and sputter coated processes. Evaporated sensors appear smooth, exhibiting very little wrinkling (although wafer-length wrinkles occasionally form around patterns during processing); the metal layer shows a regular grain pattern with spherical Au nuclei approximately 50 nm in diameter (Figure 5a). This smoothness is mirrored by the respective weakly-adhering donor (Figure 5b), which maintains a lightly speckled finish akin to the resulting metal grain. In contrast, sputtered AP sensors demonstrate wrinkled surface topography after processing that extends to the underlying PPI backing, and a continuum of elliptical Au particles (Figure 5c). The respective PDMS surface maintains the same wavy pattern after the sensor peel-off (Figure 5d). This characteristic of Au film wrinkling on PDMS is expected, though wrinkles are not apparent to the naked eye for sputtered sensors. Evaporated sensors, on the other hand, occasionally formed wafer-length wrinkles form around PPI patterns during processing; these wrinkles disappear after PPI development. Despite the differences, both instances of evaporated and sputtered AP sensors maintain their electrical interconnects and sensing properties.

### 3.3. Quantitative Comparison of TP and AP Sensors

**Figure 6:**
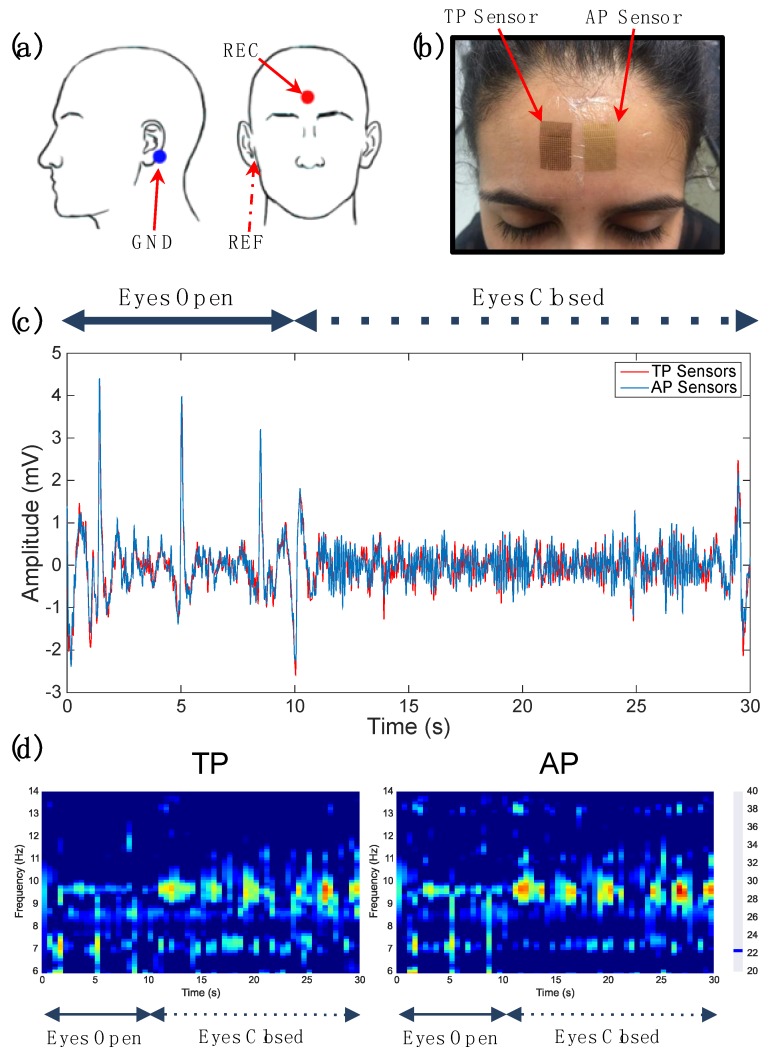
Electrophysiological comparison of sensors: (**a**) EEG testing electrode montage; (**b**) sensors applied to acquisition site—TP on the left, AP on the right; (**c**) time−voltage plot for one epoch of data: red = TP, blue = AP; and (**d**) spectrographic representation of epoch: TP on the left, AP on the right. The alpha rhythm begins just after the 10 s mark for both sensors.

To ensure functional characteristics of the electrical sensors are preserved when fabricating with the AP approach, a common eyes-opened/eyes-closed paradigm for measurement of the 8–12 Hz alpha rhythm in electroencephalogram (EEG) [48] was performed. An EEG test was chosen to demonstrate that electrical AP sensors can adequately measure lower amplitude-frequency signals, while indirectly suggesting that larger biopotentials such as ECG and EMG can be easily measured with high fidelity. This is especially true due to gold’s low impedance in high-frequency bands, as compared to bands characteristic of EEG signals [49,50]. A set of wired AP and TP sensors on Tegaderm (3M, Saint Paul, MN, USA) were connected to semi-encapsulated custom flex cabling (Pica Manufacturing, Derry, NH, USA) which is conductive to the sensors, but insulated to the skin. These sets were applied in the three-lead mastoid configuration, as per Figure 6a; each set consisted of recording (REC, forehead), reference (REF, right mastoid process), and ground (GND, left mastoid process) leads. Sensors were carefully arranged side-by-side so to prevent electrical cross-talk between channels (Figure 6b, cables not shown). AP and TP sets were wired into an Avatar EEG biopotential amplifier system (Electrical Geodesics, Eugene, OR, USA) through the flex cabling. EEG data was simultaneously recorded from both wired electrical sensor sets according to details found in the Materials and Methods section. The acquired biopotential data was sampled at 500 Hz and digitally band-passed in Matlab (MathWorks Inc., Natick, MA, USA) to a 6–14 Hz spectral range.

Voltage and time-frequency representations of data from both sensor sets are illustrated in Figure 6c,d. Time-frequency plots were generated using the robust spectrotemporal decomposition outlined in the literature [51]. In both representations, the first 10 s of data are eyes-opened followed by 20 s of eyes-closed data, which is characterized by a 10–12 Hz alpha rhythm (Figure 6c,d). Visually, it is easy to confirm that EEG data from both sensor types co-vary closely with one another. This suggests that this alternative microfabrication approach has no significantly negative effect on the acquisition capability of such electrical sensors.

Electrical AP sensor fidelity is compared to that of TP sensors by use of the Pearson’s correlation coefficient [52], which ranges from −1 to 1: −1 being negatively correlated, 1 being positively correlated, and 0 representing no correlation.

(1) $$r=\frac{\sum_{i=1}^{n}\left(X_{i}-\bar{X}\right)\left(Y_{i}-\bar{Y}\right)}{\sqrt{\sum_{i=1}^{n}{\left(X_{i}-\bar{X}\right)}^{2}}\sqrt{\sum_{i=1}^{n}{\left(Y_{i}-\bar{Y}\right)}^{2}}}$$

Equation (1) is used to calculate the r statistic for each TP-AP pair of simultaneously recorded voltage data. Table S1 (Supplementary Information) lists correlation coefficients for each of the six TP-AP trials. Many of trials resulted in similar TP and AP time series (r > 95%), suggestive of AP sensors’ high fidelity acquisition despite alternative fabrication procedures. Still, perfect correlation is not possible in that both sensors record nuances in thermal noise and motion artifact at the sight of signal acquisition.

### 3.4. Non-Electrical AP Sensors

**Figure 7:**
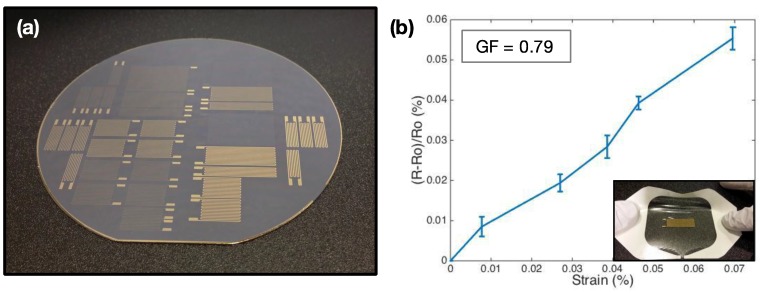
Mechanical strain sensor fabrication using AP process: (**a**) mechanical strain sensors on wafer; (**b**) characteristics of a simple mechanical strain sensor; and (**b**, **inset**) Image of AP strain sensor stretched during axial testing. Gauge factor (GF) = 0.79.

Using the AP process outlined, other sensor types are easily fabricated for monitoring through other modalities. An instance of this is the production of a variety of simple strain sensors (Figure 7a). AP mechanical strain sensors are fabricated according to the same framework presented in Figure 2; the pattern used during photolithography is replaced by the desired pattern to produce a variety of functional strain sensors with tailored gauge factors. Post-processing of these sensors follows the same peel-off process—from the donor substrate onto the receiving adhesive of choice. The axial strain-resistance performance of one AP strain sensor over several trials is illustrated in Figure 7b, and its gauge factor (GF) calculated. With a GF of 0.79, these gold strain sensors demonstrate relatively poor sensitivity to mechanical stimuli, as compared to other strain gauge varieties [53,54]. However, the emphasis here is that the present method demonstrates versatility in the types of sensors that can be produced, from which improvements can be made.

### 3.5. AP Sensors on Flexible Donor Substrate

**Figure 8:**
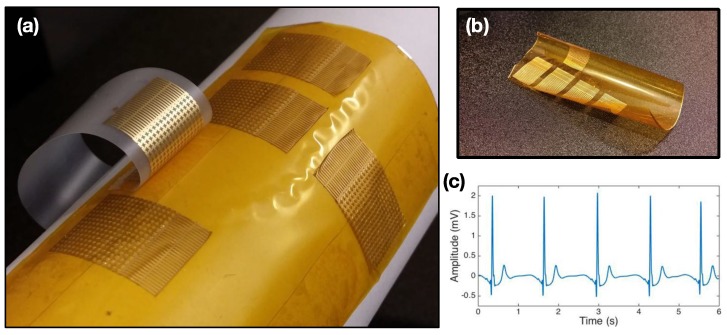
Electrical AP Sensors processed and peeled-off a flexible donor substrate: (**a**) AP sensors half-peeled by consumer tape with flexible weakly-adhering donor mounted on a tube; (**b**) free-standing flexible donor substrate with sensors; and (**c**) time−voltage plot of EKG acquired using AP sensors off flex donor.

Finally, the simple peel-off properties of this method allow us to consider fabrication on flexible donor substrates; this enables the use case of roll-to-roll post-processing. Instead of a silicon or glass wafer, Kapton film was used to fabricate electrical AP sensors; fabrication utilized the same technique outlined (Figure 8a,b). Supplemental Video S4 shows the peel-off process from the flexible Kapton-based donor. AP sensors from flexible donors show no apparent differences as compared to their analogs from rigid donor substrates. To demonstrate their function, a single-channel EKG snapshot acquired using wired AP sensors is shown in Figure 8c, clearly illustrating a strong QRS complex alongside P and T waves. One can imagine simply inserting a roll of AP sensors to an existing tape manufacturer’s production line (with some modifications), where AP sensors are directly peeled directly onto the target adhesive and sent out to the end user.

## 4. Discussion

Here we have presented a process scheme by which a class of flexible sensors can be fabricated and post-processed in a relatively efficient manner. It is important to emphasize here that while there is vast significance in flexible and stretchable sensor capability and performance, we ultimately require a process that can scale with research and consumer needs. It is mentioned that roll-to-roll processing is a ubiquitously used industrial process, which boasts efficiency at the levels of cost and automation. Rather than “re-invent the wheel” for accommodating these growing spaces, it is in our interest to leverage the maturity of other processes such as roll-to-roll, at least initially at the stage of post-processing and packaging.

To summarize, our AP sensors are fabricated on a weakly-adhering donor substrate through an inverted construction. Fabrication with PDMS is a not a new concept—previous methods have demonstrated thin film formation and encapsulation of constructs with PDMS featured as the donor during a multi-step transfer printing process. or underlying substrate [14,15,16,17]. Others have created methods by which a device is either transferred to or is initially constructed on PDMS as a final receiving substrate [55,56,57]. In some of these instances, oxidized PDMS exhibits relatively strong adhesive properties when in contact with certain materials—such as SiO_2_—that promote silanol hydrogen-bonding [58]. Presented herein, PDMS is used as a donor substrate without any modifications, so to exploit its weak (but sufficient) adhesion to Au films [59] during a one step peel-off process. In concert with the reversed deposition of Au/Cr films and polymer backing, we are allowed to simply peel-off target features, obviating the need for sacrificial layers, immersion in solvents, repetitive transfer steps, and intermediate stamps. Other fabrication details—such as the use of PPI for simultaneous patterning and polymer formation—make for a streamlined process with fewer process failures and better resource allocation during AP sensor preparation.

The use of a PPI polymer backbone is attractive here due to its electrically insulating properties, chemical resistance and thermal stability. Moreover, PPI’s known biocompatibility for clinically implantable electronic systems makes it an attractive polymer over conventional photoresists [60] Because some emphasis is placed on the use of AP sensors for wearable/clinical applications, PPI appears to be the most feasible polymer to implement in the methodology. For sensors not involving biological systems, we speculate other polymers like a photoresist can be used while maintaining the combined formation/patterning step of the AP sensor backbone, though this needs to be investigated.

Ultimately, any adhesive or adhesive-coated material that can effectively adhere to the desired pattern (for the weakly-adhering donor example, with an adhesive force approximately > 0.2 N) is likely to perform a successful peel-off of AP sensors during post-processing. Moreover, knowledge of the range of forces necessary for successful peel-off enables us to consider situations where one might initially constrain the adhesive to be used and “reverse engineer” the adhesion force between PDMS and Au films. For example, if certain types of strong adhesives cannot be used due to their damaging of a target surface (e.g., application on neonates with sensitive skin) [61], there is opportunity to tailor the PDMS–sensor interface. In doing so, one can decrease the interface adhesion for facilitating peel-off with weaker adhesive material, while maintaining AP sensor stationarity during microfabrication.

Because Au flexible sensor fabrication is possible with the weak-interface approach outlined, a next step is to better characterize the interfacial adhesion and exploit this method for use with other interfacing material types. It is known that noble metals possess a proclivity for weak adhesion to an elastomeric rubber such as PDMS [59]. We surmise the methods outlined here can be readily implemented for other metals most similar in nature to Au. Of interest might be metal substitutes such as Ag, of which can be post-processed for yielding Ag/AgCl (one of the best suited for DC coupled signal acquisition) and Pt AP sensors (used for its long-term stability and biocompatibility *in vivo*; standard practice in neural stimulation) [49,62]. AP sensors comprising Ag or Pt as the interfacing material may be possible to fabricate by simply changing the layer designations during reverse metallization.

Integrated systems of AP sensors with miniaturized back-end transmission and processing circuits may be possible using the present approach. With improved integration of active components and passive interconnections, there is opportunity to integrate with miniaturized rigid electronic circuits [63,64,65], or further transition away from rigid and stiff electronic assemblies to more flexible, stretchable, and unobtrusive options [13,15]. Advances in this regard might be spurred by recent actions to strengthen the infrastructure of U.S. manufacturing of smart flexible hybrid electronics systems [66,67].

A degree of Au film micro-scale buckling is observed during sputter-based AP sensor fabrication, though this characteristic of sputtered sensors has not been observed as detrimental to the sensor fidelity. To the naked eye, sputtered metal films on optimized PDMS maintain their adhesion during processing and do not present large wrinkles, buckling, or cracks. It is important to note, however, that an overall ease with which the sensors can be peeled-off by an adhesive is maintained in spite of this adhesion. We hypothesize that during the sputter coating process, Au nanoparticles are engulfed by the elastic PDMS surface, creating immobilized nucleation sites that resist metal film delamination during processing. Moreover, it appears that sputter coating in this instance is responsible for rendering a wavy PDMS topography—as a consequence, surface area is increased which might give rise to better adhesion. It has been reported that gaseous plasma treatment (e.g., Ar plasma) during sputter coating increases the wettability of PDMS and prevents fast hydrophobic recovery of the PDMS surface, through the creation of hydroxyl-terminated, silica-like surfaces [68,69,70]. This phenomenon, in concert with the increased surface area and engulfed Au nanoparticles, may explain the unexpected immobilization of Au films during processing on PDMS.

During early evaporation tests, buckling in Au films often lead to propagating cracks, as was expected due to differences in PDMS and Au thermal expansion coefficients. We sought to remedy the buckling issues by addressing the mechanical mismatch between the underlying PDMS and the metal film above. It is known that the relative stiffness of PDMS depends on the ratio of pre-polymer to crosslinker agents used. The result of a smaller ratio is a stiffer PDMS structure that exhibits a relatively large elastic modulus, as compared to lower ratios [71]. Keeping this in mind, stiffer concoctions of PDMS (e.g., 3:1) were used, the idea being that stiffer PDMS (of higher elastic modulus) will resist expansion, therefore reducing the disparity in expansion during thermal cycles. A reduction in film buckling was observed, with no signs of cracking in the resulting AP sensors. Moreover, total film lamination appeared to improve during processing, though this was not quantitatively inspected. Ultimately, evaporated AP sensors maintain their interconnections conductance during processing, and easily peel-off the underlying PDMS surface.

Though the AP approach allows for easy peel-off of microscale thin film features, one should consider the sensor design to be employed. Poorly fashioned sensor patterns and z-thick metal-PPI stacks may result in micro-cracks across stress raisers during the peel-off process. Other pattern characteristics, such as the x–y thickness of pattern features, may pose similar issues due to the inherent tug-of-war between the weakly-adhering donor and receiving adhesive during the peel-off process. We speculate that an increase in effective x–y area will cause a departure in the observed peel-off force threshold, requiring stronger adhesives. Conversely, a decrease in the x–y area might give rise to premature release of patterns during fabrication on the donor substrate, causing problems with device yield. Although our methodology was successful for relatively large 1 cm × 1 cm square features, design constraints and peel-off force thresholds should be characterized for better understanding the nature of this weak adhesion and applying relevant findings to future work outlined above.

Lastly, although the present method boasts efficiency over previous works, AP sensors *require* an adhesive surface for facilitating peel-off. Moreover, AP sensors cannot be re-used, as the necessary adhesive for peel-off wears down during use and is difficult to re-apply. Instead, AP sensors might be immediately suitable as single-use, peel-and-stick sensors in the arena of clinical patient monitoring—where disposable systems are preferred due to concerns of sterility and contamination—and more generally in the spaces of consumer and industrial sensing that benefit from single-use flexible and stretchable form factors.

## 5. Conclusions

In summary, the work here describes a microfabrication method utilizing unconventional procedures for faster production of ultrathin AP flexible, stretchable electrical and mechanical sensors on adhesives. AP sensor production utilizes standard microfabrication procedures while leveraging the expected weak adhesion between PDMS elastomeric substrates and thin Au films. The methodology is agnostic to the different procedures for metallization and robust to adverse cracking of sensor layers. Though sensor performance enhancement is not the aim the present method, sensors are objectively processed and peeled-off onto flexible adhesive substrates in a manner that provides substantial improvements over existing thin film methods. With significant technological advances in the realms of wearable and industrial sensors and circuits, it is important to develop and optimize methods for which production of these technologies is commensurate with their current and projected commercial demands. Keeping pace with these trends and demands will allow us to more readily disseminate flexible/stretchable electronics technology and usher in a new façade of our everyday devices.

## Supplementary Files

Supplementary File 1
