# Supplementary material for: Scalable Microfabrication Procedures for Adhesive-Integrated Flexible and Stretchable Electronic Sensors
Source: Sensors (Basel). 2015 Sep 16;15(9):23459–76. doi: 10.3390/s150923459 (PMC4610501; doi:10.3390/s150923459)
Supplement: Supplementary File 1 [file sensors-15-23459-s001.zip › sensors-15-23459-supplementary.docx]

**OPEN ACCESS**

***sensors***

**ISSN 1424-8220**

www.mdpi.com/journal/sensors

Supplementary Information

Scalable Microfabrication Procedures for Adhesive-Integrated Flexible and Stretchable Electronic Sensors. *Sensors* 2015, *15*, 23459-23476

Dae Y. Kang, Yun-Soung Kim, Gladys Ornelas, Mridu Sinha, Keerthiga Naidu and
Todd P. Coleman *

Department of Bioengineering, University of California San Diego, La Jolla, CA 92093, USA;
E-Mails: dykang@eng.ucsd.edu (D.Y.K.); ysk032@eng.ucsd.edu (Y.-S.K.); glornela@ucsd.edu(G.O.); mbsinha@eng.ucsd.edu (M.S.); agihtreek@gmail.com (K.N.)

***** Author to whom correspondence should be addressed; E-Mail: tpcoleman@ucsd.edu;
Tel./Fax: +1-858-534-8207.

1. Experimental Details

1.1. Fabrication of AP Sensors

Formation of the weak-adhering donor substrate is prepared by spin coating and curing a 50 μm thick layer of poly(dimethylsiloxane) (PDMS; Sylgard 184, Dow Corning, Midland, MI, USA) onto a 4-inch silicon or glass wafer (University Wafer, Boston, MA, USA). Thin metal films are deposited on top of the PDMS surface using either a sputter system (ATC Orion, AJA, N Scituate, MA, USA) or electron beam evaporator (Temescal BJD 1800, Ferrotec, Santa Clara, CA, USA) to form Au and Cr metal layers (in this order) of 200 nm and 5 nm in the thicknesses, respectively. A negative-tone photodefinable polyimide (HD-4104, HD Microsystems (Dupont), Wilmington, DE, USA) is spin coated at 3000 rpm for 1 min, soft baked (150 °C, 1 min), and UV-exposed (EVG620, EV Group, Sankt Florian am Inn, Austria). The polyimide layer is developed by using a modified puddle approach found in the literature [1]. The photodefined polyimide (resting on top of the wafer) is placed in a convection oven (Carbolite, Derbyshire, UK) at 250 °C for 90 min to ensure full curing of the polyimide patterns and complete bonding to the interfacing Cr layer. The wafer is then dipped in Cr and Au etchants (Transene, Danvers, MA, USA) (in this order) to remove unnecessary metal surfaces, only leaving metal layers directly under the polyimide pattern intact. Completed devices are peeled onto the target receiving substrate, such as Tegaderm (3M, Saint Paul, MN, USA), by laminating the target adhesive film over the devices and delaminating once the device is adhered. Overall process time—starting with 3 pre-PDMS donor substrates and ending with completely peeled devices from
3 donors—is approximately 5 h. Fabrication of AP sensors on flex substrate follows the same method, except that 25 μm Kapton film (DuPont, Wilmington, DE, USA) is used as the donor substrate. For TP sensors, a streamlined version of the fabrication procedures outlined in [2] were used. Under the same experimental conditions and same number of sensors, the end-to-end TP process (from unmodified donor substrate to post-transfer printing) is approximately 10 h long.

1.2. EEG & Statistical Testing

Three subjects were each tested on two separate occasions for eyes-opened and eyes-closed EEG data, acquiring data from both PMMA and AP sensors. The testing paradigm epoch is as follows: 10 s eyes-opened followed by 20 s eyes-closed. This process is repeated twice more (90 continuous seconds of EEG data, for a total of 3 epochs) to ensure data without deviant artifacts from the independent recording systems. Of these 3, the “cleanest” (*i.e.*, least noise prone) epoch is chosen per subject, per trial—this results in a total of N = 6 epochs of PMMA-AP EEG data from 3 subjects. Because both sensor sets are used simultaneously, each epoch consists of synchronized PMMA and AP biopotential data. Data sets are then post-processed to yield Pearson’s Correlation coefficient values for evaluation of similarity between PMMA and AP sensor performance.

1.3. Peel Testing—Initial Interfacial Characterization

An array of equally spaced, 1 cm × 1 cm solid square patterns were fabricated onto a weak-adhering donor substrate as outlined. Peel-off force was measured using a force gauge (M2-2, Mark-1, Minneapolis, MN, USA), rigidly coupled to a flat mount lined with adhesive tape. The adhesive end was adhered only to the full 1 cm^2^ pattern area and the force gauge pulled upwards, normal to the pattern plane. Maximum force observed during this peel-off was used to characterize the adhesion force between the PDMS-sensor interface. The process was repeated 5 times, at random positions on the donor substrate. Peel force average and variance were calculated using the data.

2. Tables

**Table S1.** Pearson’s correlation coefficients for EEG testing. TP-AP EEG test comparisons across 6 total trials.

| **Subject** | **Trial** | **Correlation Coefficient** |
| --- | --- | --- |
| 1 | 1 | 0.95 |
|  | 2 | 0.95 |
| 2 | 1 | 0.98 |
|  | 2 | 0.96 |
| 3 | 1 | 0.95 |
|  | 2 | 0.81 |

3. Videos

**Video S1. TP Process.** TP sensors are shown on a rigid donor substrate. Acetone is heated and sensors features on donor are submerged in acetone bath so to dissolve the PMMA sacrificial layer. Lift-off of sensor from donor substrate is frequently checked. Finally, a sensor is released from the underlying PMMA layer and transfer printed onto an intermediate PDMS stamp, and onto the target 3M Tegaderm substrate. “Heat Acetone” and “Submerge Features” periods run at 2000% speed and are skipped during longer periods. Total time for complete transfer = 27 min, 10 s.

**Video S2. AP Process.** AP sensors on a rigid donor substrate are ready for peel-off. An AP sensor is shown to immediately peel-off onto the target 3M Tegaderm substrate. Total time for peel-off = 35 s.

**Video S3. AP Process with Lint Roller.** AP sensors on a rigid donor substrate are ready for peel-off. A commercial-grade lint roller is used to immediately and objectively peel-off AP sensors using a simple roll technique. AP sensors peel-off completely while maintaining their pattern design and function.

**Video S4. AP Process on Flexible Carrier Substrate.** AP sensors on a flexible donor substrate are ready for peel-off. The donor substrate is flexed and bent to while carrying AP sensors. An AP sensor is shown to immediately peel-off onto the target 3M Tegaderm substrate. The AP sensor is peeled-off completely while maintaining its pattern design and function.

Acknowledgments

This research was supported by the NSF Center for Science of Information (NSF
CCF-0939370), National Science Foundation (NSF SMA-1451221), Bill and Melinda Gates Foundation (GCF-OPP1061388), Naval Medical Research Center (W911QY-12-C-0090), Gerber Foundation (GF-554-3288), Army Research Office (ARO-62793-RT-REP), The Hartwell Foundation, Kavli Foundation, National Science Foundation Graduate Research Fellowship (2012137216), and the UCSD Institute of Engineering and Medicine Graduate Fellowship.

**Conflicts of Interest**

The authors declare no conflict of interest.

References

1. Grady, M.E.; Geubelle, P.H.; Sottos, N.R. Interfacial adhesion of photodefinable polyimide films on passivated silicon. *Thin* *Solid* *Film* **2014**, *552*, 116–123.
2. Kim, D.-H.; Lu, N.; Ma, R.; Kim, Y.-S.; Kim, R.-H.; Wang, S.; Wu, J.; Won, S.M.; Tao, H.;
   Islam, A.; *et al*. Epidermal electronics. *Science* **2011**, *333*, 838–843.

© 2015 by the authors; licensee MDPI, Basel, Switzerland. This article is an open access article distributed under the terms and conditions of the Creative Commons Attribution license (http://creativecommons.org/licenses/by/4.0/).
